# 

**Published:** 1847-10

**Authors:** 


### AMERICAN JOURNAL

OF

### Dental Science.

PUBLISHED UNDER THE AUSPICES OF THE

### American Society of Dental Surgeons.

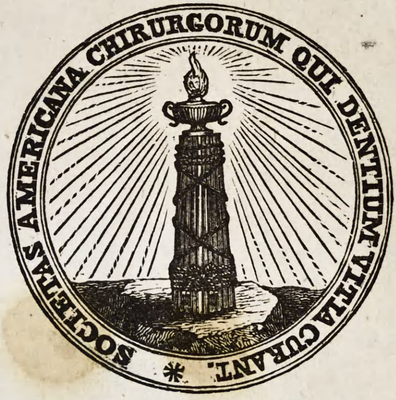

VOLUME VIII.—1847-8.

EDITORS:

CHAPIN A. HARRIS, M. D., D. D. S.
AMOS WESTCOTT, M. D., D. D. S.
WILLIAM H. DWINELLE, D. D. S.

BALTIMORE:
ARMSTRONG & BERRY,
PHILADELPHIA: LINDSAY & BLAKISTON.
JOHN W. WOODS, PRINTER.

*To Subscribers in England.*—The fourth number of the last volume of the Journal, which should have been in Liverpool by the 1st of July, was regularly boxed and sent to an express office to be taken to Boston, and sent out by one of the British steamers, but owing to some carelessness on the part of one of the agents, it was detained for several weeks in Philadelphia, and finally shipped on board of a packet ship, which sailed in August last. We shall endeavor, for the future, to prevent any accident of this kind.—*Balt. Ed.*

*Omission.*—We are compelled, for want of room, to omit several articles and notices which we had designed publishing in the present number. They shall appear in our next.—*Balt. Ed.*

